# Effectiveness of an evidence-based care pathway to improve mobility and participation in older patients with vertigo and balance disorders in primary care (MobilE-PHY2): study protocol for a multicentre cluster-randomised controlled trial

**DOI:** 10.1186/s13063-022-07017-x

**Published:** 2023-02-06

**Authors:** Caren Horstmannshoff, Stefanie Skudlik, Jenny Petermann, Theresia Kiesel, Tobias Döringer, Alexander Crispin, Joachim Hermsdörfer, Juliane Köberlein-Neu, Klaus Jahn, Stefan Schädler, Petra Bauer, Karen Voigt, Martin Müller

**Affiliations:** 1 Centre for Research, Development and Technology Transfer, Rosenheim Technical University of Applied Sciences, Hochschulstr. 1, 83024 Rosenheim, Germany; 2 Department of Sport and Health Sciences, Chair of Human Movement Science, Technical University of Munich, Georg-Brauchle-Ring 60/62, 80992 Munich, Germany; 3 Faculty of Medicine Carl Gustav Carus, Technische Universität Dresden, Fetscherstr. 74, 1307 Dresden, Germany; 4 Institute for Medical Information Processing, Biometry, and Epidemiology, Ludwig-Maximilian University of Munich, Marchioninistr. 15, 81377 Munich, Germany; 5 Center for Health Economics and Health Services Research, University of Wuppertal, Rainer-Gruenter-Str. 21, 42119 Wuppertal, Germany; 6 German Centre for Vertigo and Balance Disorders, Ludwig-Maximilian University of Munich, Marchioninistraße 15, 81377 Munich, Germany; 7 Schoen Clinic Bad Aibling, Kolbermoorer Str. 72, 83043 Bad Aibling, Germany; 8 Physiotherapie im Schloss, Schloss 88, 3454, Sumiswald, Switzerland; 9 Faculty of Applied Health and Social Sciences, Rosenheim Technical University of Applied Sciences, Hochschulstr. 1, 83024 Rosenheim, Germany; 10 Department of Primary Care and Health Services Research, Medical Faculty, Heidelberg University, Im Neuenheimer Feld 130.3, 69120 Heidelberg, Germany

**Keywords:** Dizziness, Vertigo, Balance disorder, Complex intervention, Primary care, Care pathway, Multicentre cluster randomised controlled trial, Mobility, Physiotherapy

## Abstract

**Background:**

Vertigo, dizziness or balance disorders (VDB) are common leading symptoms in older people, which can have a negative impact on their mobility and participation in daily live, yet, diagnosis is challenging and specific treatment is often insufficient. An evidence-based, multidisciplinary care pathway (CPW) in primary care was developed and pilot tested in a previous study. The aim of the present study is to evaluate the effectiveness and safety of the CPW in terms of improving mobility and participation in community-dwelling older people with VDB in primary care.

**Methods:**

For this multicentre cluster randomised controlled clinic trial, general practitioners (GP) will be recruited in two regions of Germany. A total of 120 patients over 60 years old with VDB will be included. The intervention is an algorithmized CPW. GPs receive a checklist for standardise clinical decision making regarding diagnostic screening and treatment of VDB. Physiotherapists (PT) receive a decision tree for evidence-based physiotherapeutic clinical reasoning and treatment of VDB. Implementation strategies comprises educational trainings as well as a workshop to give a platform for exchange for the GPs and PTs, an information meeting and a pocket card for home care nurses and informal caregivers and telephone peer counselling to give all participants the capability, opportunity and the motivation to apply the intervention. In order to ensure an optimised usual care in the control group, GPs get an information meeting addressing the national guideline. The primary outcome is the impact of VDB on participation and mobility of patients after 6 month follow-up, assessed using the Dizziness Handicap Inventory (DHI) questionnaire. Secondary outcomes are physical activity, static and dynamic balance, falls and fear of falling as well as quality of life. We will also evaluate safety and health economic aspects of the intervention. Behavioural changes of the participants as well as barriers, facilitating factors and mechanisms of impact of the implementation will be investigated with a comprehensive process evaluation in a mixed-methods design.

**Discussion:**

With our results, we aim to improve evidence-based health care of community-dwelling older people with VDB in primary care.

**Trial registration:**

DRKS, DRKS00028524 retrospectively registered on March 24, 2022.

**Supplementary Information:**

The online version contains supplementary material available at 10.1186/s13063-022-07017-x.

## Background

Vertigo, dizziness and balance disorders (VDB) are common leading symptoms in older patients and can be caused by various conditions [1, 2]. The prevalence is about 20% in people over 60 and 30% in people over 70 and increases with age [1, 3, 4]. Cases of VDB are among the 20 most common reasons for encounter in general practitioners’ (GP) practice in Germany [5, 6] and can have a highly disabling impact on the daily lives of older adults and are associated with gait abnormalities, an increased risk of falls, social withdrawal, limitations in autonomy [1, 7, 8], depression and reduced functional ability [9–12]. In primary care, the diagnosis of VDB is often inadequate, the according management partly ineffective or insufficient [13–16]. Current treatment approaches include patient education, pharmaceutical therapy, medication review and physiotherapy (PT) [17]. Recent evaluations indicate that physiotherapy in particular can have a beneficial impact on balance and fall risk for many patients with VDB [2, 18–23]. Despite that, the current clinical guideline for German primary care practitioners recommends the prescription of physiotherapy for patients with acute dizziness only in fairly limited cases [17].

To improve primary care of community-dwelling older patients with VDB, we developed and piloted an evidence-based, multidisciplinary care pathway (CPW) [24–26]. Findings from the pilot study indicate that benefits for patients are possible, both GPs and PTs were satisfied with the educational trainings and patients responded well to the physiotherapy. However, recruitment was challenging.

## Methods

This study protocol follows the Standard Protocol Items: Recommendations for Interventional Trials (SPIRIT) Checklist [27] for Trials. The whole project is structured according to the UK Medical Research Council guidance for development and evaluation of complex interventions [28]. In the previous study, we covered the two steps ‘Development and Feasibility/ Piloting’ [24, 25]. In the current study, we proceed with the step, ‘Evaluation’.

The study is designed as a pragmatic, controlled, multicentre, cluster-randomised, cluster blinded, trial with two parallel groups and a 1:1 allocation and takes place in two regions in Germany: South-eastern Bavaria and Saxony. Both sites have an urban centre with a rural environment.

### Objectives

#### Primary objective

The main objective of the present study is to evaluate the effectiveness and safety of the CPW in terms of improving mobility and participation in community-dwelling older people with VDB in primary care in comparison to a control group, which receives optimised usual care. The impact of VDB on mobility and participation will be assessed by the Dizziness Handicap Inventory (DHI) questionnaire [29] as primary outcome.

#### Secondary objectives

Secondary outcomes are physical activity, static and dynamic balance, falls and fear of falling as well as quality of life. We will also evaluate the safety in terms of falls. Cost-effectiveness and cost-utility of the CPW will be assessed in a health economic evaluation alongside the trial. To understand the behaviour change process, as well as barriers and facilitating aspects of implementation, the study will be accompanied by a comprehensive process evaluation.

### Eligibility criteria

#### Patients

Primary target population are community-dwelling older patients aged at least 60 years, who consulted their GP with recent or chronic complaints of VDB. They should be able to stand up on their own and stand for two minutes with support.

Exclusion criteria for patients are:

- Not able to give written informed consent,
- A DHI less than 12 points [30]. A DHI of at least 12 points is required, since the suggested minimal clinical important difference is 12 points [30] and patients with a lower DHI score are not at risk to reach the effectiveness threshold.
- Moderate to severe cognitive impairments as defined by the Mini-Mental State Examination of less than 20 points [31],
- The presence of psychiatric disorders identified by International Statistical Classification of Diseases and Related Health Problems (ICD) codes (F10.-F19, F20.-F29, F30., F31., F32.2, F32.3, F32.8, F32.9, F33.2, F33.3, F33.8, F33.9),
- Limited life expectancy (≤ 1 year) due to an advanced disease with a poor prognosis estimated by GP,
- VDB caused by current substance abuse,
- Unable to complete questionnaires and follow instructions because of insufficient command of the German language.

#### Health professionals

Participating GP practices, PT practices and home care nursing services are required to have an accreditation of the German statutory health insurance. GP practices with practice software that is not eligible for identification patients as potential study participants will be excluded.

### Sample size

Sample size calculation was informed by data of a cohort study with the same population [32]. Data gave evidence of an ICC = 0.04 and an expected standard deviation of 25. A cluster size of 10 is assumed, i.e. the variance inflation factor is 1 + (10 − 1) × 0.04 = 1.36. The study plans to detect a score difference between intervention groups of at least 12 points [30] on a two-sided alpha level of 0.05 with a power of 80%, resulting in a total sample size of ((1.96 + 0.84)^2^ × 2 × 25^2^ × 1.36)/12^2^ = 92.6. Recruitment of 12 clusters à 10 patients accounts for a dropout rate of 23%. The study will be conducted in two waves, resulting in six clusters (three per study centre) à ten patients being recruited per wave. Current experience shows that a few clusters will possibly fail to recruit the necessary number of patients. In this case, we will recruit more patients in other clusters to compensate for this. To maintain the statistical power of the study, the following rule will be applied: number of patients to be additionally recruited in other clusters = 1.5 × number of patients that some clusters fail to recruit.

### Recruitment

**Fig. 1:**
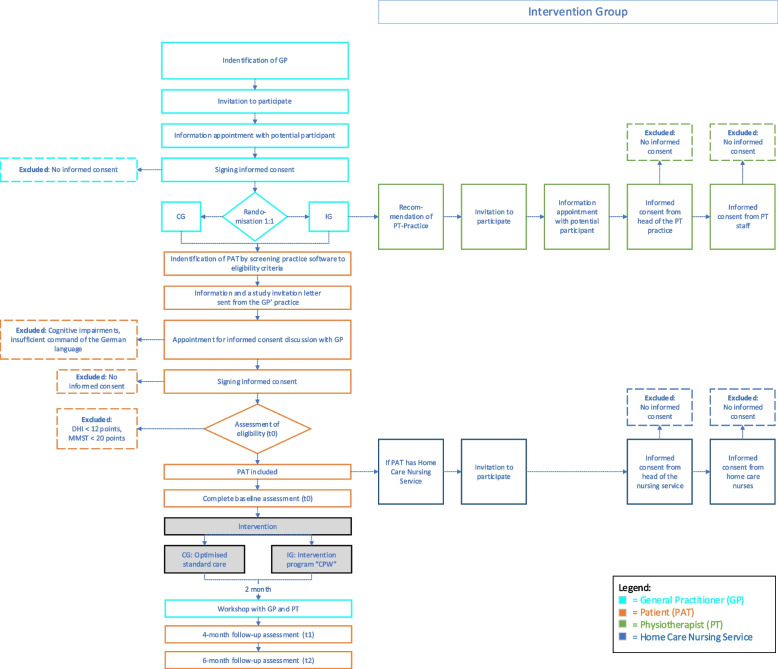
Recruitment and flow of all participants

Every potential study participant, patients, GPs and additionally in the intervention group PTs and home care nurses, will receive written and verbal study information and will be required to give written informed consent. The identification and recruitment of clusters (GP practice and their patients), PTs and home care nurses are presented (see Fig. 1).

In a first step, GP practices will be identified by searching GP registers in each study region. Cooperation networks will be consulted and the project will be presented to professional associations. Included GPs will then select potential patients as participants by searching their data bases for relevant ICD Codes (see Table 1) within the past 3 years. The GP will send a study invitation letter to eligible patients. Interested patients will make an appointment for the informed consent discussion with the GP. If included patients in the intervention group receive home care, their home care nursing services will be informed about the study. The director of the home care service will be asked to select at least two nurses, who are in contact with the participating patient at least than once a week, to attend the educational training (see Implementation strategies). PT practices will be identified through asking included GPs for existing cooperation and through regional searches. Identified PT practices will receive an invitation letter. If interested, the head of the PT practice can select up to two PTs to attend the educational training.

**Table 1:** ICD Codes for having recent or chronic complaints of VDB

ICD codes	Explanation
F45.8	Other somatoform disorders
G43.1	Migraine with aura
G62	Other and unspecified polyneuropathies
G63	Polyneuropathy in diseases classified elsewhere
H81	Disorders of vestibular function
I95.1	Orthostatic hypotension
R26 (without R26.1)	Abnormalities of gait and mobility
R29.6	Repeated falls
R42	Dizziness and giddiness

As incentives, participating GP practices receive a case payment per patient (60 €). Participating PT practices will receive a case payment per patient (30 €), and home care services will receive a training on the ‘National Expert Standard in Long Term Care on mobility’ [33] after the study.

### Allocation

GP practices will be randomly allocated to the intervention or control group after written informed consent. Stratified block randomisation by region (two times three clusters in Saxony, two times in south-eastern Bavaria) will be used. The block length will be two, ensuring an equal number of clusters providing the experimental and the control intervention, respectively. The most recent version of the Statistical Analysis System SAS (SAS Institute, Cary, NC, USA) will be used for generating the sequence. Allocation will not and cannot be concealed from the members of the research team distributing the study materials to the care providers. The allocation sequence will be generated by a statistician who is not involved in the study.

### Intervention

#### Experimental intervention

The intervention is an algorithmized evidence-based multidisciplinary CPW to enhance the care of older persons with VDB and thus promote their mobility and participation in daily living. It illustrates all steps of the patient pathway through a standardised approach consisting of two main components:

1. A paper-based *checklist for standardise clinical decision making*, which contains evidence-based diagnostics, treatment and referral options for the whole treatment period and is applied by previously trained GP. The aim of the checklist is to help the GP to make a classifying diagnosis, to guide the next steps and to prioritise them.
2. A *decision tree* for evidence-based physiotherapeutic clinical reasoning and treatment of VDB. It consists of recommendations and options for anamnesis (including information about clinical pattern), specific assessments, treatment and evaluation. The decision tree will be applied by previously trained PT, if the treating GP prescribes physiotherapy.

**Fig. 2:**
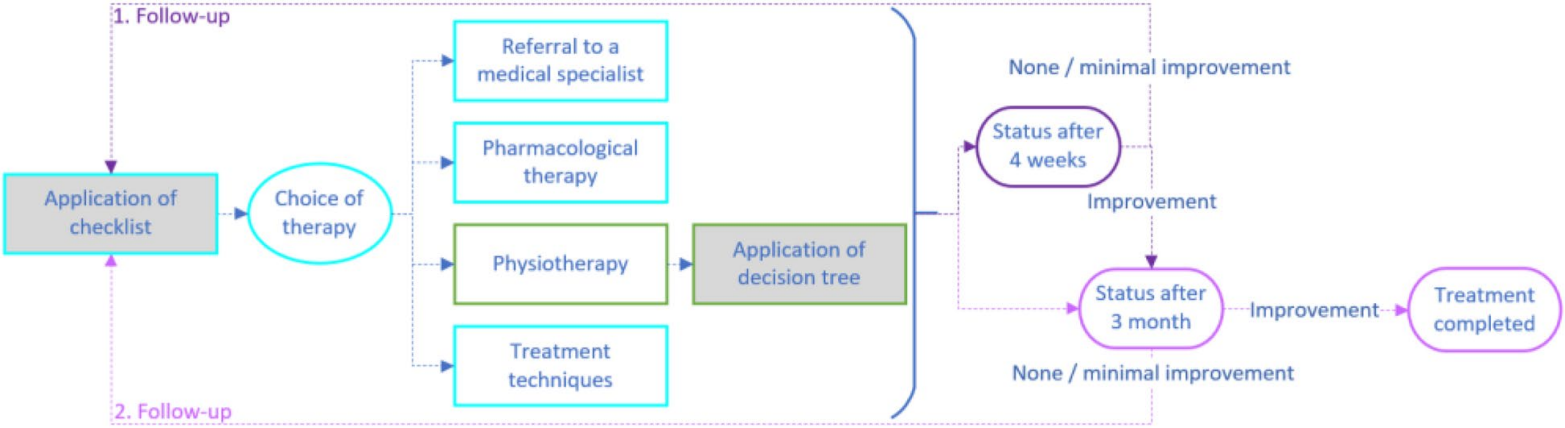
Intervention flow

At the beginning of the 4-month intervention period, the patients make an appointment with their GP who performs the anamnesis and the examinations based on the checklist. The anamnesis involves questions regarding the quality, character, frequency and duration of VDB and accompanying symptoms. Then, further examinations like position manoeuvre and neurological tests will be undertaken by the GP. Depending on the results of the anamnesis and examinations, the checklist contains suggestions for further referral and treatment (see Fig. 2). Referral suggestions can be the referral to a neurologist, otorhinolaryngologist, internist/cardiologist and PT. Treatment options may include symptomatic bridging therapy with medication or general treatments such as medication adjustment. The treatment by and therefore the referral to a trained PT is recommended when the checklist/GP comes to the following conclusions: the patient suffers from benign paroxysmal positional vertigo, other factors requiring examination by another physician specialist can be excluded and/or has taken place before with no results.

The trained PT will use the decision tree for clinical reasoning and planning and applying the treatment. According to the decision tree, the PT will use systematic recommendations and options for a physiotherapeutic anamnesis, which leads to specific hypotheses. Following the algorithm, the PT is then able to differentiate and choose the right physiotherapeutic assessment. Based on the results, the decision tree leads him/her to a specific hypothesis and corresponding evidence-based treatment options (the main focus is on vestibular rehabilitation) including educational material for the patient, like home exercise programs and flyers with information. Last step will include the evaluation and documentation of the chosen therapy.

At the follow-up-visits after 4 weeks and 3 months, the GP will use the checklist again. In case of worsening of symptoms or 8 weeks PT without improvement, a referral to a medical specialist is recommended. In the event of a slight improvement in symptoms, PT is recommended for a further 4 weeks. If there is significant improvement after 4 weeks, only the second follow-up examination is suggested. In the event of significant improvement after 3 months, the treatment is successfully completed within the context of the study. A modification of the intervention is not planned. There are no restrictions regarding concomitant treatments and interventions during the trial.

#### Control group

To avoid performance bias, we aim to keep GPs and patients masked regarding group allocation. Therefore, we provide an active control intervention for the control group cluster (GPs and their patients). It consists of a 30-min online educational training on the German national guideline ‘acute dizziness’ for GPs [17], which is held by a GP.

#### Logic model

The logic model (see Additional file 1) illustrates the relationship between the planned work (‘resources’ and ‘activities’) to implement the intervention (implementation strategies), the expected mechanism of impact (based on a behaviour change theory), the intended results (realised intervention with ‘output’, ‘outcome’ and ‘impact’) and categories of possible influencing factors. The structure of the logic model follows the Logic Model Development Guide [34]. As behaviour change theory, we applied the capability, opportunity and motivation behaviour (COM-B) model originating from the behaviour change wheel [35] as theoretical foundation for a structured development of implementation strategies and there with to support the intended behaviour change for both, the health professionals and the patient. The basic assumption is that a behavioural change of health professionals is a prerequisite for a change in patients’ health behaviour. The use of resources and the improvement of these through certain activities (e.g. knowledge enhancement through training) influence the capability, motivation and opportunities, which ultimately alter the behaviour of the health professionals. This can lead to a behaviour change at the patient level, e.g. by passing on the gained knowledge to patients, thus influencing their COM-B. As a result, behaviour change can contribute to an improvement in mobility and participation of older patients with VDB. For classifying and identifying influential factors on implementation, the five main elements of Consolidated Framework of Implementation Research are used [36].

### Implementation strategies

To reach the study target, various implementation strategies will be delivered (see Table 2). Some of these strategies have been tested in the pilot study and according adaptations were made [25].

**Table 2:**
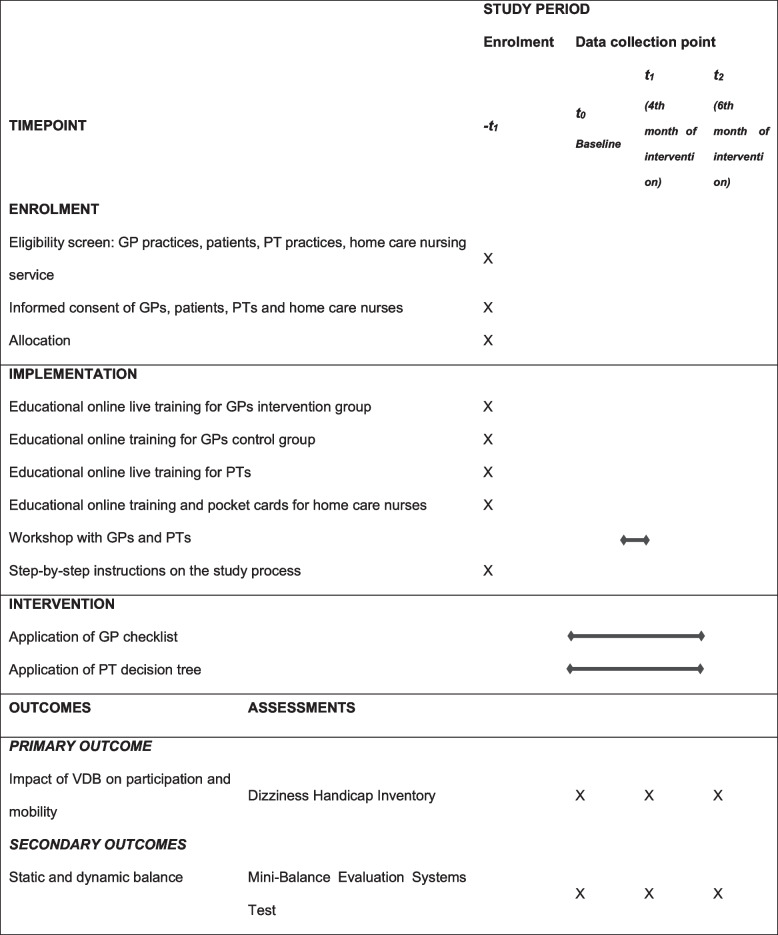

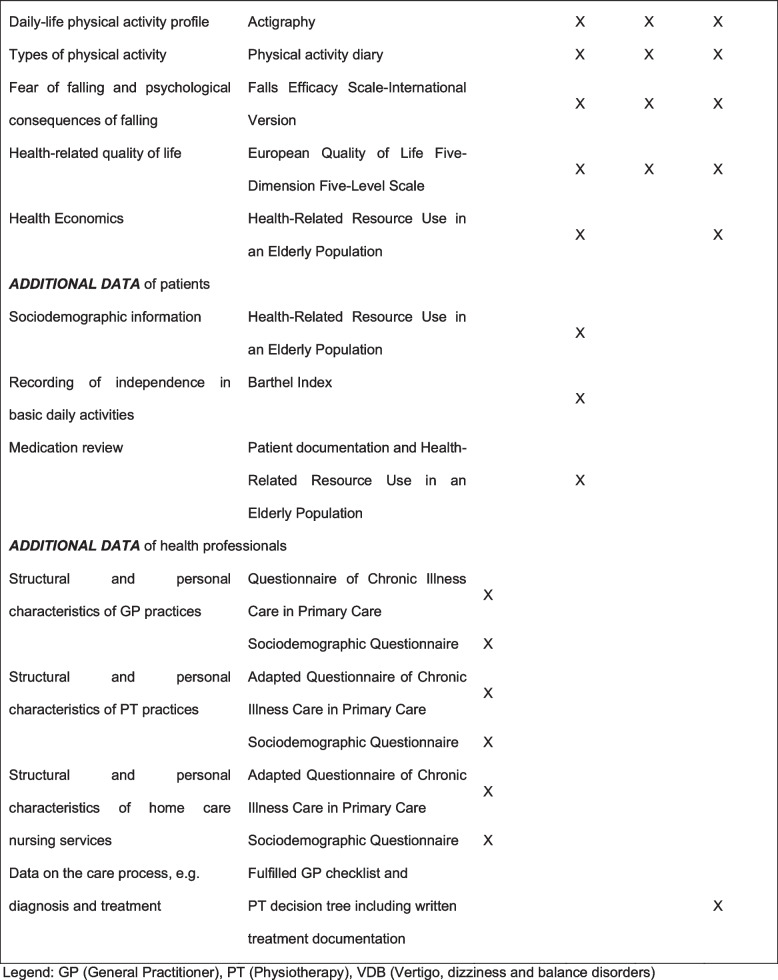
Schedule of enrolment, interventions, research outcomes and assessments according to SPIRIT

*GP* general practitioner, *PT* physiotherapy, *VDB* vertigo, dizziness and balance disorders

In the intervention group, GPs and if requested their medical assistants will participate in a two hours lasting educational training. The training will be held by a VDB expert and neuro-otologist and will include an update on recent diagnostic and therapeutic developments for VDB in older patients, as well as an introduction into the use of the checklist for diagnostic screening. The PTs will receive a 1-day educational training called ‘Dizziness and/or balance disorders in the vestibular rehabilitation of older patients in primary care’ which enables the participating PTs to understand the procedure according to the decision tree and enables them to apply it. To make sure, that the PTs can transfer their knowledge into practice, there will be the opportunity so share treatment videos and receive feedback from the expert. Both events will be held live online. After 8 weeks, GP practices and PTs will be invited to a 1-h online moderated follow-up workshop to exchange experiences about the study and ideas to improve the communication in an interdisciplinary team working in primary care. A pocket card for the home care nurses serves as a reminder on their role as supporters of the patients’ adherence to the intervention (reminders on potential physiotherapeutic exercises, making and keeping appointments, filling out the exercise diary, fall prevention measures). In addition, they will receive a training about VDB in older people, fall prevention and their role in the study. All participants will receive step-by-step instructions on the study process. Moreover, there will be a telephone hotline for all participant groups to answer their questions on content and methodology and to respond to problems or needs in a timely manner.

### Outcomes

The primary and secondary outcome will be assessed on patient level in the intervention group and control group at three measurement points: at baseline before the intervention (*t*_0_), after 4 months (*t*_1_) and after 6 months (*t*_2_). Additional data on GP practices, PT practices and home care nursing services level will be assessed at *t*_0._ For an overview of the research outcomes and assessments, see Table 2.

#### Primary outcome measures

##### Dizziness handicap inventory

The impact of VDB on participation and mobility of patients will be assessed using the Dizziness Handicap Inventory (DHI). It is a condition-specific instrument to assess self-perceived disability, such as limitations in mobility and activity and participation in older patients [37]. The DHI contains 25 items of three different subscales: Functional subscales (nine questions), emotional subscales (nine questions) and physical subscales (seven questions). The patients will rate their problems on a scale ranging from ‘no’ (0 points) and ‘sometimes (2 points) to “yes’ (4 points). The total score ranges from 0 points to 100 points, while the higher the score, the greater the self-perceived handicap. A mean decrease of 12 points between *t*_0_ and *t*_2_ is considered as a clinically meaningful difference [30]. For our study, we will use the Dizziness Handicap Inventory—German Version (DHI-G). It has high reproducibility and good internal consistency [29, 37].

#### Secondary outcome measures

##### Physical activity

The main secondary outcome is the qualitative and quantitative change in daily-life physical activity measured by wearable sensor-based actigraphs from movisens [38]. In addition, patients will be asked to fill in a physical activity diary about their activities of daily living and occurrence of VDB.

##### Safety

Falls during the intervention period could be a possible risk or complication. The incidence of falls during the intervention period as main safety outcome will be documented in the physical activity diary. Additionally, falls within the last 6 months before the start of the intervention will be assessed.

##### Falls efficacy scale—international version

We will examine concerns to falls during physical activities and the social dimension of fear of falling using the German version of the Falls Efficacy Scale—International Version, which consists of 16 items [39]. The German version has a high internal and test-retest reliability [40].

##### Mini-balance evaluation systems test

We will investigate static and dynamic balance by using the validated German Version of the Mini-Balance Evaluation Systems Test [41]. It has four domains: anticipatory proactive balance, reactive postural control, sensory orientation and dynamic gait and 14 items.

##### European quality of life five-dimension five-level scale

The health-related quality of life will be assessed by the German version of the European Quality of Life Five-Dimension Five-Level Scale (EQ-5D-5L). The five dimensions are mobility, self-care, usual activities, pain/discomfort and anxiety/depression. The validity and reliability are assessed as sufficient to be used in economic evaluation studies [42].

##### Health-related resource use in an elderly population

Healthcare resource utilisation and medication will be assessed using the validated ‘Questionnaire for Health-Related Resource Use in an Elderly Population’ (Fragebogen zur Inanspruchnahme medizinischer und nicht-medizinischer Versorgungsleistungen im Alter). It consists of 14 items and is a valid tool to determine health related resource use in older population [43].

#### Additional data sources

To describe the sample and potential confounder, additional data will be assessed: Sociodemographic information (age, gender, family status) for descriptive purposes, the Barthel Index [44, 45] to assess the independence in basic daily activities, the Mini Mental Status Test [31, 46] to measure cognitive impairment and the Questionnaire of Chronic Illness Care in Primary Care [47] to assess structural and personal characteristics from GP practices and an adapted version for PT practices and home care nursing services.

### Process evaluation

To not only assess whether a complex intervention is effective but to understand how and under which circumstances the intervention is working, a process evaluation is an essential part of testing complex interventions [48]. Therefore, we will monitor the whole study process with a process evaluation, which is based on our logic model (see Additional file 1).

The process evaluation follows the Medical Research Council guidance for complex interventions [28] along with an adapted version of Grant’s framework for designing process evaluations of cluster-randomised trials [49]. We aim to shed light on essential aspects of the process, including the recruitment and reach of participants, the delivery of the intervention to and response of all participants including implementation. In addition, mechanisms of impact and context will be examined [48]. To thoroughly monitor the expected behaviour change (see Additional file 1), as well as the other factors mentioned above, we will use a mixed-methods approach (Table 3). Individual interviews will be conducted at *t*_2_ with all GPs and with the half of PT practices and group interviews with 20% of the home care nursing services. We will also conduct interviews with about 15 patients of the intervention group and 15 of the control group at timepoint *t*_0_ and *t*_2_. The interviews of t_0_ will focus on the treatment of VDB before the study and the attitude towards the intervention.

**Table 3:** Schedule of process evaluation according to SPIRIT

	Study period				
	Enrolment	Data collection point			Close-out
**Timepoint**	***-t***_***1***_	***t***_***0***_	***t***_***1***_	***t***_***2***_	***Post t***_***2***_
**Treatment pre study**					
Interviews with patients		X			
**Recruitment and reach**					
Contact protocol	X				
Cancellation forms	X				
Individual interviews with all participants		X		X	
**Delivery**					
Standardised evaluation forms for the educational trainings including supportive materials	X				
Implementation protocol	X				
Field notes via checklist/decision tree				X	
Field notes of contact via telephone or email		X	X	X	
Interviews with all participants				X	X
**Response**					
Field notes of contact via telephone or email		X	X	X	
Field notes via checklist/decision tree				X	
Interviews with all participants				X	X
Evaluation of the telephone helplines					X
**Unintended consequences**					
Field notes of contact via telephone or email		X	X	X	
Field notes via checklist/decision tree				X	
Interviews with all participants					X
**Context**					
Interviews with all participants				X	
**Data collection procedures and organisational aspects**					
Field notes by the study assistant afterwards each measurement appointment		X	X	X	
Interviews with all participants					X

### Health economic evaluation

The objective of the health economic evaluation is to estimate the cost-effectiveness of the intervention in terms of additional costs per additional patient who experienced a decrease of 12 points on the DHI questionnaire between *t*_0_ and *t*_2_. Moreover, a cost-utility analysis will consider the additional costs per additional quality-adjusted life year (QALY).

The health economic evaluation will be performed from a societal perspective. Therefore medical and non-medical resource utilisation will be measured for retrospective periods of 6 months at baseline and at *t*_2_ using the Health-Related Resource Use in an Elderly Population. For monetary valuation of resource utilisation, unit costs will be calculated for all services and goods (privately purchased or prescribed) in Euro.

### Data collection

Data will be collected in form of self-filled paper-and-pencil questionnaires and in form of physical activity assessments by research assistants who are blinded to group allocation and will go through a profound assessment training program. Success of blinding will be estimated by asking the research assistants to guess the allocation of the study groups after every measurement and comparing the answers with what would be expected by chance [27]. Unblinded assessors will be replaced by another trained, blinded researcher whenever possible. Otherwise, loss of blinding will be recorded and considered in the analysis. Data collection with patients will take place in the patients’ home. Only pseudonymised data will be collected and every cluster, PT and home care nurse will receive a pseudonymised code. Interviews with all participants will be conducted by telephone and tape-recorded.

### Data management

We created a concept for data protection in cooperation with both data protection officials in both study sites that is based on the EU General Data Protection Regulation (EU-GDPR) and federal legislation. Only the responsible researchers will have access to the pseudonym data sets. The data will be encrypted and password-protected when being transferred between secure servers. Data managers and biostatisticians will receive the original paper-based pseudonymised questionnaires. Two research assistants of their team will check the data for inconsistency and completeness and then entered into a database and validated through double entry so that the biostatistician can analyse data without having access to information about the allocation. The pseudonymised data will be handled and stored according to the data protection declaration of the institution. Tape-recorded qualitative data from interviews will be transcribed verbatim and afterwards the audio file will be deleted.

### Data analysis

The data analysis of the measured outcomes will be done by a blinded biostatistician who is not involved in the study. The primary analysis will be based on a linear mixed model of the DHI changes between t_0_ and t_2_, using intervention group and initial DHI as fixed factors and a random intercept for cluster. The statistical inference will be based on the Wald *t*-test for the intervention on an alpha level of 5% (two-sided). Similar models will be applied for exploratory analyses of the secondary outcomes. No pre-specified subgroup analyses are planned. Due to the restricted sample size, no additional adjustments for other fixed effects are planned. Range and cross-checks of the outcome variables and key baseline patient characteristics will be performed. Impossible or implausible data will trigger queries to the team of the principal investigator. All analyses will be based on the intention-to-treat principle. In case of dropouts, the last available observation will be carried forward. No interim analyses are planned.

For the process evaluation, quantitative data will be analysed descriptively. Qualitative data from interviews and field notes from research team, GPs and PTs will be analysed with content analysis [50], which will be performed by two independent researchers from the study centres.

To analyse the cost-effectiveness of the intervention, an incremental cost-effectiveness ratio and an incremental cost-utility ratio will be calculated as the ratio of the difference in mean cost and the difference in mean health effects (DHI or QALY) between study groups during the 6-month follow-up period. Non-parametric bootstrap method will be employed to generate confidence intervals around the difference in mean cost and the difference in mean health effects. Uncertainty surrounding the cost-effectiveness ratio and cost-utility ratio will be presented on the cost-effectiveness plane. Furthermore, cost-effectiveness acceptability curves, based on the incremental net-monetary benefit will be constructed. The study will be terminated when all collected data have been analysed and utilised within the analysis plan.

### Harms

In order to monitor the unintended consequences of the study, we will stay in contact with the GPs, and PTs have implemented a telephone hotline for all participant groups, review the notes on the checklists, decision trees and physical activity diary and conduct interviews with patients.

Additionally, an advisory board, consisting of members of the MobilE-Net Network, will supervise the trial.

## Discussion

Due to the experiences gained from our pilot study, we have critically and carefully revised our recruitment, intervention and implementation strategies. First, the recruitment options are expanded in terms of the use of various approaches (e.g. involvement of local networks, use of faxes, call for participation via website and newspaper). Medical assistants are incorporated in the whole study process, as they are the primary point of contact with the practice and relationships with practice staff are considered as essential for maintaining commitment to the study [51, 52]. The adherence to the intervention protocol by the GPs and PTs will be improved by using a revised version of the checklist and decision tree, revised educational trainings (e.g. more hands-on exercises), the provision of supportive materials and a study folder with all relevant study documents. Team work between different health professionals is considered as a key to deliver high quality primary care [53]. To establish interdisciplinary team working in primary care, communication is a main facilitator [54]. In order to stimulate the interdisciplinary exchange between GPs and PTs, we will perform a workshop after 8 weeks to share experiences and ideas. To improve the patients’ adherence and motivation even in the absence of informal caregivers, we involve home care services which receive a pocket card containing reminders on relevant study topics (e.g. filling physical activity diary). In addition, patients will be assisted by the study team in filling in the questionnaires and the physical activity diary. The physical activity diary and the corresponding manual were revised to make it more understandable and easier to be filled in for the target group. The assessment of physical activity behaviour by the International Physical Activity Questionnaire [55] and the use the actigraphy device StepWatch™ resulted in missing values or seemed not to be appropriate for our target population as reported in other studies [56, 57]; thus, it would not be used in the present study anymore.

The aim of the present study is to evaluate the effectiveness of this complex intervention and to understand the change processes. We expect a behaviour change at cluster and individual level in the intervention group and thus a more effective management of VDB and an improvement in the quality of care, which in turn leads to improvements in the mobility and participation of older community-dwelling adults with VDB.

If the efficacy of the intervention has been proven successful, a refinement of the intervention is planned according to the recommendations of the evaluation study and then strive for transferring it into standard care. To realise this, we are cooperating with a regional statutory health insurance.

## Trial status

Protocol version 1.1, 07.12.2022. The study started in June 2020 and the recruitment of clusters began in October 2021. The first cluster was included in December 2021, and the last cluster will be included in January 2023. Recruitment was stopped between January and March 2022 due to rising incidence of COVID-19 infections and the high burden on GP practices in Germany. Since the length of the recruitment stop was not predictable and a relevant change in study procedures was considered, we decided not to publish the study protocol until the situation became clearer again.

## Supplementary Information

**Additional file 1. **Logic model.

## Data Availability

The GP checklist and decision tree will be made available on reasonable request after the termination of the study. The consent materials are available from the corresponding author on request.

## Abbreviations

COM-B: Capability, opportunity and motivation behaviour
CPW: Care pathway
DHI: Dizziness Handicap Inventory
GP: General practitioner
ICD: International Statistical Classification of Diseases and Related Health Problems
PT: Physiotherapist
SPIRIT: Standard Protocol Items: Recommendations for Interventional Trials
QALY: Quality-adjusted life year
VDB: Vertigo, dizziness or balance disorders
