# Supplementary figures and images for: Effectiveness of an evidence-based care pathway to improve mobility and participation in older patients with vertigo and balance disorders in primary care (MobilE-PHY2): study protocol for a multicentre cluster-randomised controlled trial
Source: Trials. 2023 Feb 6;24:91. doi: 10.1186/s13063-022-07017-x (PMC9902065; doi:10.1186/s13063-022-07017-x)

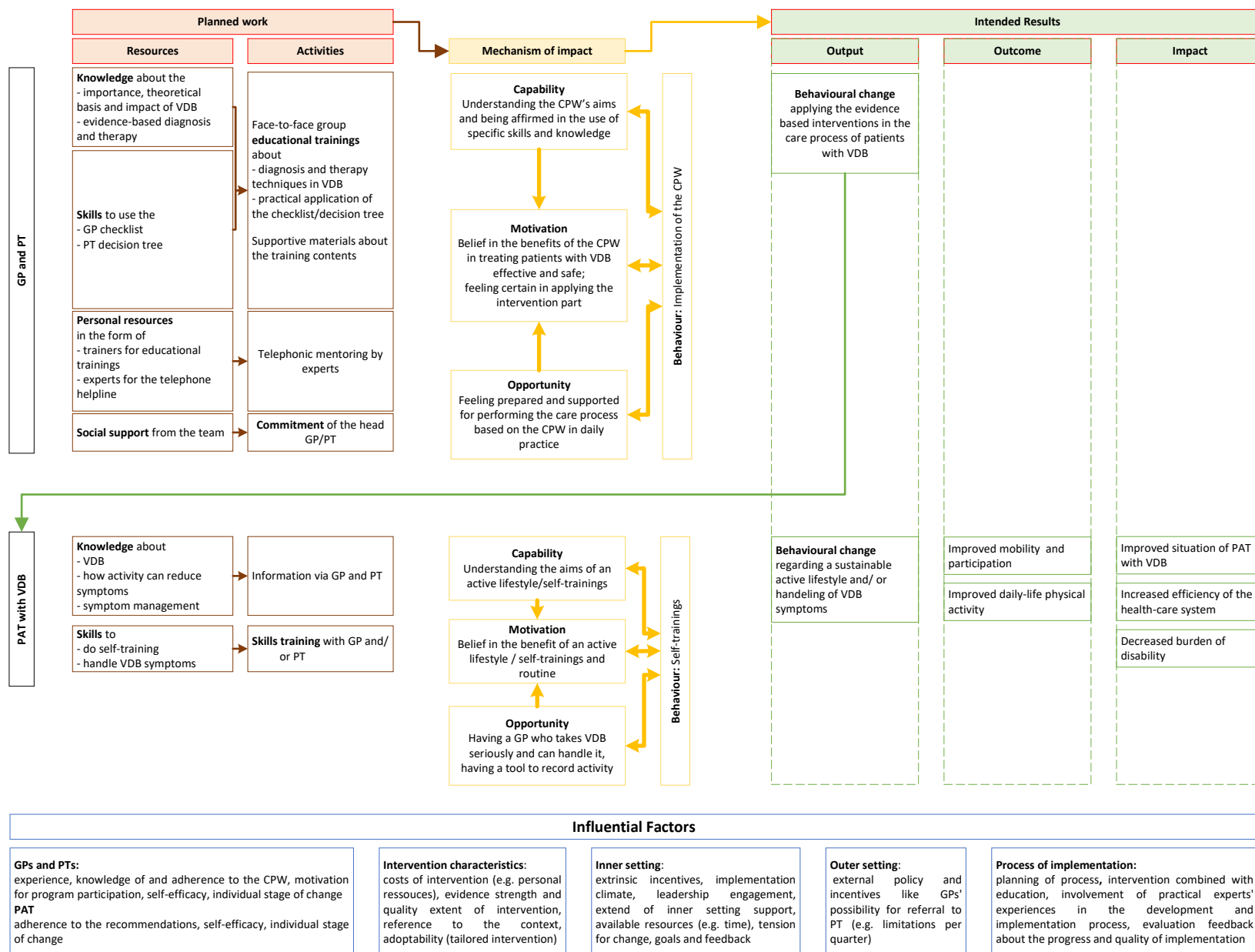

Supplement: Supplementary file 1 — Additional file 1. Logic model. [file 13063_2022_7017_MOESM1_ESM.pdf]
